# Mycosporine-Like Amino Acids and Marine Toxins - The Common and the Different

**DOI:** 10.3390/md20080008

**Published:** 2008-05-22

**Authors:** Manfred Klisch, Donat-P. Häder

**Affiliations:** Department for Biology, Friedrich-Alexander University, Staudtstr. 5, 91058 Erlangen, Germany; E-mails: mklisch@biologie.uni-erlangen.de; dphaeder@biologie.uni-erlangen.de

**Keywords:** mycosporine-like amino acids, marine toxins, algae, cyanobacteria

## Abstract

Marine microorganisms harbor a multitude of secondary metabolites. Among these are toxins of different chemical classes as well as the UV-protective mycosporine-like amino acids (MAAs). The latter form a group of water-soluble, low molecular-weight (generally < 400) compounds composed of either an aminocyclohexenone or an aminocyclohexenimine ring, carrying amino acid or amino alcohol substituents. So far there has been no report of toxicity in MAAs but nevertheless there are some features they have in common with marine toxins. Among the organisms producing MAAs are cyanobacteria, dinoflagellates and diatoms that also synthesize toxins. As in cyclic peptide toxins found in cyanobacteria, amino acids are the main building blocks of MAAs. Both, MAAs and some marine toxins are transferred to other organisms e.g. via the food chains, and chemical modifications can take place in secondary consumers. In contrast to algal toxins, the physiological role of MAAs is clearly the protection from harmful UV radiation by physical screening. However, other roles, e.g. as osmolytes and antioxidants, are also considered. In this paper the common characteristics of MAAs and marine toxins are discussed as well as the differences.

## Introduction

Natural products from marine organisms have attracted increasing research interest in recent years [1,2]. The number of newly reported marine compounds increased steadily over the last decades up to almost 4000 new compounds described between 2001 and 2005 [2]. A wide variety of products from marine algae are of high actual or potential economic impact. Polyunsaturated fatty acids, carotenoids and phycocolloids are examples for marine products used in the food industry, while other compounds serve as templates for the development of new pharmaceutical drugs [3].

**Figure 1:**
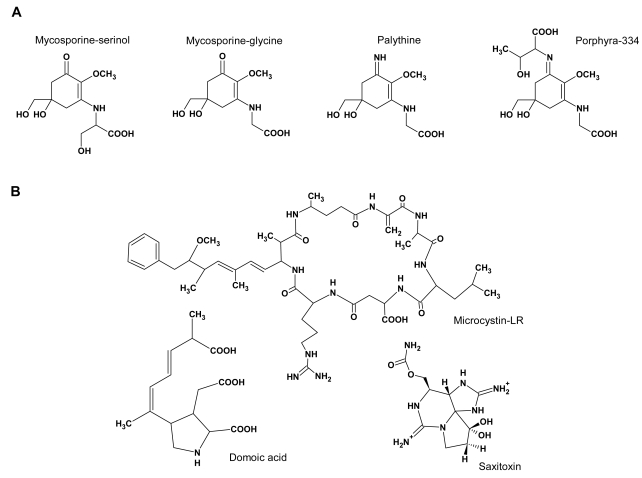
Structures of some typical MAAs and some important algal/cyanobacterial toxins. A: The fungal mycosporine mycosporine-serinol, and algal/cyanobacterial MAAs mycosporine-glycine (an oxo-MAA) and the imino-MAAs palythine and porphyra-334. B: The marine toxins microcystin-LR, produced by toxic cyanobacteria, domoic acid, predominantly produced by diatoms, and saxitoxin produced by toxic dinoflagellates and some cyanobacteria.

A remarkable group of marine natural products are the mycosporine-like amino acids (MAAs). An outstanding characteristic of these compounds is their high UV absorption with molar absorptivities (ɛ) of around 40 000 l mol^−1^ cm^−1^ (e.g. [4]). MAAs are water-soluble, low molecular-weight (generally <400) compounds composed of either an aminocyclohexenone or an aminocyclohexenimine ring, carrying nitrogen or amino alcohol substituents (see Fig. 1A) [5]. They are found in a wide variety of marine, freshwater and to a smaller degree in terrestrial organisms [6]. There is limited evidence that MAAs are derived from early steps of the shikimate pathway [7,8]. However, the biochemical pathway of MAA synthesis is still largely unknown, as well as its genetic base. The most primitive organisms capable of MAA synthesis are cyanobacteria (apart from one single bacterial species [9]). Among eukaryotic life, MAAs are synthesized in algae, fungi and lichens (symbioses between algae or cyanobacteria and fungi). Animals, which are lacking the shikimate pathway, but nevertheless contain MAAs in their body, are believed to derive these compounds either from their food or from symbiotic algae or cyanobacteria [10,11]. Recently this view has been challenged by the report of genes enconding enzymes of the shikimate pathway in the genome of a marine animal [12].

The function of MAAs in phototrophic organisms is primarily the protection of the organisms from deleterious UV radiation [13–15], but additionally they may function as compatible osmolytes [16] or as antioxidants [17]. Due to their high photostability MAAs are interesting candidates as UV sunscreens in cosmetic formulations [18,19]. Other UV absorbing compounds found in nature include scytonemins [20,21] and usnic acid [22]. The occurrence of these compounds is much more restricted than that of MAAs: Scytonemins are found only in cyanobacteria and cyanobacterial lichens [21,23] and usnic acid only in lichens [24]. Similar to MAAs, the synthesis of scytonemins and usnic acid is triggered by UV radiation and UV protection is an important function of these compounds [22,25]. In spite of these similarities, this review will focus on a comparison between MAAs and marine toxins.

Marine toxins have a tremendous economical impact due to their deleterious effects [26]. Poisonings by marine toxins can be grouped according to the chemical structures of the causative toxins and main symptoms of intoxication [26]: **1:** Paralytic shellfish poisoning (PSP), caused by a group of closely related tetrahydropurine compounds including saxitoxin (STX), the first chemically characterized PSP toxin (see Fig. 1 B). **2:** Diarrheic shellfish poisoning (DSP), caused by heat-stable polyether and lipophilic compounds isolated from various species of shellfish and dinoflagellates. **3:** Domoic acid (see Figure 1 B) causing amnesic shellfish poisoning (ASP) sometimes also referred to as domoic acid poisoning. **4:** Neurologic shellfish poisoning (NSP), caused by brevetoxins, tasteless, odourless, heat and acid stable, lipid soluble, cyclic polyether neurotoxins produced by the marine dinoflagellate *Karenia brevis* (formerly known as *Gymnodinium breve* or *Ptychodiscus brevis* [27]. **5:** Azaspiracid shellfish poisoning, caused by azaspiracids. **6:** Ciguatera fish poisoning (CFP), caused by ciguatera toxins, lipid soluble polyether compounds that are formed by biotransformation in the fish of precursor gambiertoxins. These poisonings in humans are caused by the ingestion of shellfish or fish that has accumulated toxins from their food. The primary producers of the aforementioned toxins are eukaryotic algae, mainly dinoflagellates. Only amnesic shellfish poisoning is caused by a diatom [28].

In freshwater environments, the main producers of toxins are prokaryotic cyanobacteria. The most frequently found cyanobacterial toxins in blooms from fresh and brackish waters are the cyclic peptide toxins of the microcystin (see Fig. 1 B) and nodularin families. Microcystins have been found in planktonic *Anabaena*, *Microcystis*, *Oscillatoria* (*Planktothrix*), *Nostoc* and *Anabaenopsis* species and in terrestrial members of the *Hapalosiphon* genus. Nodularin has been reported only from *Nodularia spumigena* [29]. Other cyanobacterial toxins are neurotoxins, including saxitoxin and analogues, anatoxin-a(S), an organophosphorous compound that acts as a potent irreversible acetyl cholinesterase inhibitor, and anatoxin-a [30].

To our knowledge there are no studies published addressing toxicity of MAAs. However, it seems likely that adverse effects, if there were any, would have been found empirically due to the widespread use of MAA-containing organisms as food, such as the red macroalga *Porphyra* sp. [31], terrestrial cyanobacteria of the genus *Nostoc* [32] and shellfish [33], without indications of adverse effects caused by MAAs. Nevertheless, the distribution of MAA synthesis and toxin production in a number of organisms leads to the question of possible relations between these groups of bioactive compounds.

## Functions of MAAs and toxins

Although there are more possible functions of MAAs in an organism, the role that is assumed primarily is the protection from deleterious UV radiation. Apart from antioxidant functions that have been reported exclusively for mycosporine-glycine [34,35] possible other roles of MAAs in desiccation tolerance as well as heat and cold tolerance are far less corroborated [36]. Merely circumstantial evidence for the UV-protective role of MAAs has been derived from the correlation of the MAA concentration and the UV radiation received by organisms. UV exclusion experiments showed that MAA concentrations in corals decline when grown under exclusion of UV radiation [37]. Numerous studies have shown correlations of MAA concentrations with gradients of UV radiation caused by water depth (e.g. [38]), UV transparency of water and altitude of the lakes (e.g. [39]) or latitudinal gradients [40]. Greater susceptibility to UV radiation was found in corals from deep water as compared to those from shallow water [38] and the photoinhibition of photosynthesis by UV radiation is decreased in dinoflagellates with high MAA concentrations induced by culture under high light conditions as compared to those with low MAA concentrations that were grown under low light. This protective effect was found, as could be expected, most pronounced in the wavelength range of maximal absorption by MAAs, i.e. between 310 and 360 nm [15]. The dinoflagellate *Gyrodinium dorsum* is able to tolerate at least twice as high doses of UV-B radiation before its motility is inhibited when it has been induced to synthesize MAAs by moderate UV-A radiation beforehand [13].

The UV-protective effect of MAAs is derived from their strong UV absorption with high molar absorptivities, their high photostability and the ability to dissipate the absorbed energy as heat. The photostability of MAAs is very high. The quantum yield of photodecomposition of the MAAs porphyra-334 and shinorine are 2.4 10^−4^ and 3.4 10^−4^, respectively [41]. In addition to the screening of cellular compounds from UV radiation, MAAs might also quench excited states of thymine by nonradiative energy transfer, and prevent the formation of photoproducts [42]. There is, however, no evidence for the importance of this mechanism *in vivo*.

Osmoprotective functions of MAAs might be concluded from their high abundance in organisms from hypersaline environments [16]. Highest MAA concentrations are generally found in organisms from marine or saline environments, but MAAs do not reach concentrations that could balance the osmolarity in the medium, so that additional compatible solutes, such as glycine-betaine or others, are required [36]. The possible involvement of MAAs in osmotic regulation was demonstrated in experiments in which the salinity of the medium was reduced by dilution with fresh water: the MAAs were rapidly excreted to the medium in amounts proportional to the degree of dilution [16].

An additional function of mycosporine-glycine may lie in the protection from oxidative stress. Whereas imino-MAAs having an cyclohexenimine core, like shinorine and porphyra-334, are not prone to oxidation, the oxo-MAA mycosporine-glycine that is constituted by an cyclohexenone core ring (see Fig. 1 A), shows a moderate antioxidant activity [17] and is an effective quencher of singlet oxygen generated by photosensitization [35]. The antioxidant activity has not only been demonstrated *in vitro*, but also *in vivo*: the coral *Stylophora pistillata*, containing only small amounts of mycosporine-glycine was found significantly more prone to heat-induced oxidative stress than *Platygyra ryukyuensis* containing a 20-fold higher concentration of mycosporine-glycine. Concomitant with the exposure to oxidative stress, a decline in the concentration of mycosporine-glycine was found in both species [34]. Singlet oxygen quenching activity *in vitro* has also been demonstrated for mycosporine-taurine but evidence for an antioxidative role *in vivo* is not available so far [43].

MAAs, although slightly different in their substituents, are a homogenous group of chemical compounds showing strong UV absorption. Marine toxins, in contrast are a structurally diverse group with the common denominator of toxicity. Some of the functions of marine toxins are connected with this characteristic, such as allelopathic effects [44] and the defence against herbivores [45]. It has been shown that microcystin-LR may exert a phytotoxic effect on aquatic macrophytes [46] resulting in growth inhibition, reduction in photosynthetic oxygen production and changes in pigment patterns. Detrimental effects of microcystin-RR on other freshwater phytoplankton species have been reported, with growth inhibition, chlorosis, changes in carbohydrate and protein content, among other symptoms [47]. However, no phytotoxic effects of microcystin-LR have been found in the aquatic macrophyte *Lemna gibba* [48].

An example of the strong effect of some toxins on grazers has been demonstrated, e.g., in experiments with the toxic dinoflagellate *Prorocentrum lima*, that produces okadaic acid and nauplii of the shrimp *Artemia salina. Artemia* cysts incubated in the cell-free culture medium of *P. lima* hatched, but mortalities were recorded for nauplii that hatched in, and metanuaplii exposed to, test solutions (autoclaved filtered seawater plus cell-free medium) that contained at least 50% of the cell-free medium. Animals exposed to cells of *P. lima* readily fed on the cells. Some nauplii, especially those just hatched, ingested only one cell before dying, while three days old nauplii ingested up to six cells before dying [45]. Even if the effect of toxins is not lethal to the grazer, toxic species may be avoided. In feeding experiments, the copepod *Acartia tonsa* was allowed to choose between toxic dinoflagellates (*Alexandrium minutum*), containing either low or high (up to 2.5 times more) amounts of PSP-toxins, and non-toxic alternative prey. The ingestion of the more toxic dinoflagellates was reduced while the ingestion of the non-toxic alternative remained unchanged [49].

The toxin domoic acid (DA) plays a role in the uptake of nutrients (Fe and Cu) and possibly the mitigation of Cu toxicity by acting as a chelating agent. The compound is released to the surrounding medium by toxin-producing diatoms under iron-limiting conditions. Fe acquisition improves markedly when DA is artificially added to the medium as well as the effect of Cu toxicity is reduced [50].

## Co-occurrence of MAAs and toxins in cyanobacteria and eukaryotic algae

MAAs are reported from almost all algal taxonomic groups examined so far, however, highest concentrations are found in rhodophytes, dinoflagellates, cryptomonads and raphidophytes [51–53]. There are few studies addressing the presence of MAAs and toxins in aquatic organisms at the same time (e.g. [51]). Nevertheless, a general pattern of co-occurrence of MAAs and toxins in certain groups can be observed.

### Cyanobacteria

The occurrence of MAAs is widespread among cyanobacteria, and there are numerous toxin producers in this very old group of microorganisms. Therefore, even if there is a lack in specific studies dealing with UV-absorbing compounds and toxin production simultaneously, it can be postulated that co-occurrence of MAAs and toxins is frequent. The bloom-forming cyanobacterium *Microcystis aeruginosa* was found to produce the MAAs Porphyra-334 and shinorine, but the presence of toxins in the strain used in the experiments was not tested [54]. Despite the fact that the term ‘microcystin’ is derived from the genus *Microcystis*, by far not all *Microcystis* isolates are toxic. In a study conducted on cyanobacteria from a German lake, individual *Microcystis* colonies were selected under a microscope, identified and screened for the presence of a gene involved in mycrocystin biosynthesis (mcyB). Most colonies (73%) of *Microcystis aeruginosa* contained the mcyB gene, whereas only 16% of the colonies assigned to *M. ichthyoblabe* and no colonies of *M. wesenbergii* showed the mcyB gene [55]. Another toxin producing genus of cyanobacteria is *Nodularia*. Not all *Nodularia* species are toxic, and toxin production seems to occur in the planktonic species *Nodularia spumigena*, while benthic species are nontoxic [56]. *Nodularia spumigena* occurs worldwide and is responsible for toxic cyanobacterial blooms e.g. in the Baltic Sea [57]. Three *Nodularia* species from the Baltic Sea (*N. spumigena*, *N. baltica* and *N. harveyana*) were found to produce the MAAs porphyra-334 and shinorine upon UV-B irradiation [58]. The filamentous bloom-forming cyanobacterium *Aphanizomenon flosaquae* is marketed today as a food supplement and has been used as a source for the preparation of porphyra-334 [19]. However, strains of this species have been shown to produce PSP toxins, among them saxitoxin [59].

### Dinoflagellates

An extensive screening for the presence of UV-A- and UV-B-absorbing compounds in cultured microalgae (206 strains from 152 species) showed an especially high abundance of UV-absorbing compounds among dinoflagellates, including members of the genera *Alexandrium*, *Amphidinium*, *Gymnodinium*, *Heterocapsa*, *Karlodinium*, *Kryptoperidinium*, *Peridinium*, *Prorocentrum*, *Scrippsiella*, *Symbiodinium* and *Woloszynskia* [53].

Carreto *et al.* [51] investigated the composition of pigments, MAAs and PSP toxins (saxitoxin and analogues) in the toxic dinoflagellates *Alexandrium tamarense*, *A. catenella* and *A. minutum*. These bloom forming dinoflagellates accumulate high concentrations of MAAs in varying composition. Toxicity and toxin composition varied widely among the *Alexandrium* isolates analysed. The toxin contents of *A. tamarense* and *A. catenella* were similar (63.2 and 50.5 fmol cell^−1^) and several times higher than those of *A. minutum* (1.0 fmol cell^−1^). Other studies typically concentrate either on MAAs or toxins.

Although not all dinoflagellates produce toxins, many members of this group are notorious for their toxin production. These are often associated with the phenomena commonly called “red tides”. This term can be quite misleading, because many toxic blooms occur when waters are not discolored while other blooms in which the high biomass and specific pigments of the dinoflagellates turn the water red are not toxic [60]. Blooms of harmful microalgae are now called harmful algal blooms or HABs. Documentation of HABs has expanded greatly over the last few decades, and many efforts are undertaken for better detection by modern techniques [61]

### Other algae

The marine toxin domoic acid is produced by diatoms of the genera *Pseudonitzschia*, *Amphora* and *Nitzschia*. It was first isolated from the red macroalga *Chondria armata* and later also found in the red macroalgae *Chondria baileyana*, *Alsidium corallinum*, *Amansia glomerata*, *Digenea simplex* and *Vidalia obtusiloba* [62]. Occurrence of MAAs was reported from *Pseudonitzschia* [63] and *Nitzschia* [64], and from a member of the genus *Chondria* (*Chondria arinata*) [65].

## Influence of environmental factors on toxin and MAA production

### Effects of radiation

MAA production is primarily stimulated by UV radiation of different wavelength ranges. UV-induced synthesis of MAAs has been reported from cyanobacteria, eukaryotic phytoplankton and macroalgae. In the non toxic dinoflagellate *Gyrodinium dorsum*, MAA synthesis is induced mainly by radiation around 310 nm, but also considerably by UV-A wavelengths [66]. In the red macroalga *Chondrus crispus* a monochromatic action spectrum of MAA synthesis showed maximal effects in the UV-A with peaks at 320, 340 and 400 nm [67]. In cyanobacteria UV-B wavelengths are most effective [68,69]. Experiments employing light/dark cycles indicate that in cyanobacteria and algae MAAs are synthesized during the light period. In the rice field cyanobacteria *Anabaena* sp., *Nostoc commune* and *Scytonema* sp. that produce mainly shinorine, a bisubstituted MAA, under natural solar radiation increasing concentrations were found only during the light periods, whereas almost constant values were found at the beginning and the end of the dark period [70]. DCMU, an inhibitor of the photosynthetic electron transport, blocked the synthesis of MAAs in the dinoflagellate *Alexandrium excavatum*. From this was concluded that MAA synthesis is closely linked to photosynthesis [71]. However, in the cyanobacterium *Chlorogloeopsis* PCC 6912 MAA synthesis can be induced in the absence of PAR under osmotic stress or artificial UV radiation [68]. Further evidence that photosynthesis is not in all cases essential for the biosynthesis of MAAs comes from the fact that mycosporines are synthesized by fungi [72], and the single case of a nonphotosynthetic bacterium that is able to produce shinorine, a bisubstituted MAA [9].

Synthesis of toxins such as cyanobacterial microcystins may also be influenced by light. In *Microcystis aeruginosa*, microcystin is synthesized nonribosomally via a multifunctional enzyme complex, consisting of both peptide synthetase and polyketide synthase modules coded for by the mcy gene cluster. Both mcyB and mcyD (parts of mcy coding for peptide synthetase and polyketide synthase modules) transcript levels were increased under high light intensities and red light. Blue light and certain artificial stress factors (methylviologen and NaCl) led to reduced transcript amounts. There appeared to be two light thresholds, between dark and low light (16 μmol of photons m^−2^ s^−1^) and between medium (31 μmol of photons m^−2^ s^−1^) and high light (68 μmol of photons m^−2^ s^−1^) at which a significant increase in transcription occurred [73].

### Effects of nutrients

There are pronounced effects of nitrogen supply on the synthesis of MAAs. Nitrogen-limited cultures of the marine dinoflagellates *Akashiwo sanguinea* (syn. *Gymnodinium sanguineum*) and *Gymnodinium* cf. *instriatum* showed a dramatic decrease in their MAA content when the NO_3_^−^ concentration in the medium was reduced from 833 μmol to 25 μmol (more than threefold) or 5 μmol (more than sixfold). This decrease was not uniform in all MAAs with mycosporine-glycine, an oxo-MAA (containing only one N atom per molecule in contrast to two N atoms in other MAAs) decreasing least [74]. In the red macroalga *Porphyra columbina*, addition of ammonium (50 μM or 250 μM) to the medium combined with different irradiation treatments led to increased MAA concentrations based on dry weight. The MAA concentration was stimulated most in combination with UV-A plus PAR radiation (29 %) [75]. In the cyanobacterium *Anabaena variabilis*, application of ammonium to the medium leads to the induction of MAA synthesis in the absence of UV radiation (unpublished observation). Not much is known on the influence of nutrients other than nitrogen on the synthesis of MAAs.

There are also major effects of nutrients on toxin production. In cultures of the toxic dinoflagellate *Alexandrium minutum*, the production of PSP toxins was greatly enhanced under P-limiting conditions as long as the N supply was sufficient. In stationary-phase cultures the toxin content per cell was greatly increased (more than eightfold) under high N:P ratios (1000:5) as compared to lower N:P ratios. Experiments showed that sufficient protein stocks in the cells are necessary for this dinoflagellate to accumulate high amounts of PSP toxins [76]. As for MAAs, also for the production of PSP toxins the form of N supply has a strong effect. In *Alexandrium tamarense*, ammonium induced the highest concentration of intracellular toxin, followed by urea and then nitrate. The toxin content was dependent on the cellular N status of nitrate grown cells only, suggesting that the competition for N in toxin production with other metabolic pathways such as growth may be different among N sources [77].

In the diatom *Pseudonitzschia multiseries*, production of domoic acid (DA) was enhanced by Fe deficiency and by Cu toxicity. During the exponential phase there was a significant inverse function of cellular growth rates and domoic acid production per cell. Addition of DA to the medium of non toxic diatom strains under Fe deficiency or Cu stress enhanced the uptake of Fe or alleviated the toxicity of Cu, which is in good agreement with the function of DA as a chelating agent [50]. Also under Si and phosphate limitation DA synthesis is increased, and inversely correlated with growth rate [78–80].

### Other factors

In some cyanobacteria MAAs also act as osmoprotectants and are consequently induced by osmotic stress [68]. An influence of temperature on MAA synthesis has also been proposed, because MAA concentrations in field samples correlate with the habitat temperature. However, these temperature differences are coupled to higher incident solar radiation. Experimental evidence on temperature effects is scarce [11]. In contrast to UV and salt stress, neither increased temperature stress nor cold shock induced MAA formation in the cyanobacterium *Chlorogloeopsis* PCC 6912 [68].

Whereas the role of optical radiation, especially UV, as the primary elicitor of MAA synthesis is grounded on overwhelming evidence, a similar main factor for toxin synthesis is absent. In addition to nutrient effects, domoic acid production in *Pseudonitzschia* species may be enhanced by the pH of the surrounding medium. This might, in addition to nutrient limitation, contribute to increased DA production during diatom blooms [81].

The presence of grazers can induce synthesis of toxins such as gonyautoxins in the dinoflagellate *Alexandrium minutum*. Waterborne cues from a copepod (*Acartia tonsa*) induce toxin production in this harmful algal bloom-forming dinoflagellate. Induced *A. minutum* contained up to 2.5 times more toxins than controls and was more resistant to further copepod grazing [49].

## Distribution of MAAs and algal toxins in food webs

Marine toxins are transferred through the food chain from the algae producing them to animals feeding on them. Important sources of intoxication in humans are edible shellfish species. During the process of filtration the dinoflagellate cells and cysts are transported to the oesophagus and the stomach of the bivalve molluscs. The digestion takes place in the stomach and the diverticulae. The PSP toxins are released and enter the digestive organs. The particular toxin mixture retained in soft tissues of the shellfish varies in concentration and over time, and is determined by the species and strains of the dinoflagellates and shellfish as well as by other factors like environmental conditions [26]. But also other marine animals such as copepods are able to take up PSP toxins from their food and potentially transmit them to zooplankton-grazing fish, as suggested by high toxin concentrations in the latter [82].

MAAs are produced by algae and cyanobacteria, but they are also found in corals and other marine life forms including invertebrate and vertebrate animals (e.g. [83]). As the shikimic acid pathway is the supposed biosynthetic origin of MAAs, these compounds are presumably acquired either by the diet or by endosymbiotic algae [11]. The dietary acquisition and accumulation of MAAs from the diet was confirmed experimentally in the green sea urchin *Strongylocentrotus droebachiensis* maintained on a controlled diet of the marine red alga *Mastocarpus stellatus*, rich principally in the MAA shinorine [84]. MAAs were accumulated specifically in the ovaries of this organisms, while in other tissues no differences between the animals fed on a MAA-rich diet and the control animals were found. Embryos of this sea urchin from adults fed an MAA-rich diet showed a significant MAA concentration-dependent protection from UVR-induced cleavage delay compared with those from urchins fed a diet lacking MAAs [14]. Similar feeding experiments were conducted with marine crustaceans species, the amphipod *Amphitoe valida* and the isopod *Idothea baltica*, from the mid-littoral of the Patagonia coast (Argentina). These showed a species-specific accumulation behavior in the two organisms. Fed on a diet rich in MAAs, *A. valida* accumulated higher concentrations of MAAs as compared to *I. baltica*, and *A. valida* with high MAA concentrations was found more resistant to UV-B radiation than the same organism with low MAA concentrations [85]. The ability to acquire MAAs from food has also been tested in medaka fish *(Oryzias latipes)* and in mice, and it was found that the teleost fish was able to acquire MAAs from the diet and accumulate them specifically in the eye, while the mammal was not [86].

## Parallels in the biosynthesis of MAAs and marine toxins

**Figure 2:**
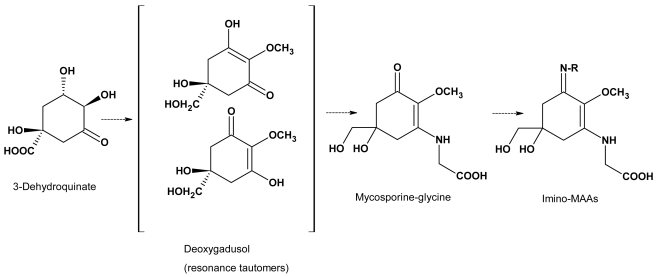
Proposed reaction scheme for the biosynthesis of MAAs, starting from 3-dehydroquinate via deoxygadusol and the oxo-MAA mycosporine-glycine to imino-MAAs. The reactions involve reduction of the carboxylic group of 3-dehydroquinate, methylation of the hydroxyl-group at C4 and attachment of one (mycosporine-glycine) or two amino acids or amino alcohols (bisubstituted MAAs such as shinorine or porphyra-334).

Although details of the biosynthesis of MAAs in marine algae and phototrophic symbioses remain to be demonstrated, it is commonly assumed that they are derived from early steps of the shikimate pathway. Favre-Bonvin *et al.* [7] suggested that the shikimate pathway intermediate, 3-dehydroquinate (DHQ), is the precursor for the six-membered carbon ring common to fungal mycosporines (Fig. 1). Synthesis of fungal mycosporines and of MAAs presumably proceeds from DHQ via gadusols (cyclohexenones) (Figure 2) [6]. Other natural compounds containing cyclohexenone rings are ketocarotenoids such as echinenone and canthaxanthin. Echinenone is produced from β-carotene by the introduction of a keto-group into the β-ionone ring (for a review see e.g. [87]). In the mycotoxin griseofulvin from *Penicillium griseofulvum*, radioactively labeled acetate is incorporated in the cyclohexenone ring structure [88]. In contrast to this neither in the ring structure of fungal mycosporines [7] nor in that of cyanobacterial MAAs [89] radioactively labelled acetate is incorporated. Thus it can be assumed that in spite of similar structural features MAA biosynthesis differs fundamenally from ketocarotenoid and griseofulvin biosynthesis.

As for MAAs, the biosynthetic pathways of many marine toxins are not fully understood. Microcystin biosynthesis has been first elucidated in two *Microcystis* strains [90–92]. The mcy gene cluster in *Microcystis* encodes six multienzymes that can be assigned to the family of nonribosomal peptide synthetases (NRPS) and type I polyketide synthases (PKS-I), respectively. Nonribosomal peptides (NRP) and polyketides are a structurally diverse group of secondary metabolites that have been identified in a number of prokaryotes and lower eukaryotes [93].

The structure of the cyanobacterial cyclic peptides are obviously much more complex than those of MAAs, but nevertheless, they share the use of amino acids (or amino alcohols, in the case of some MAAs) as building blocks. The multitude of mycosporines and MAAs is mainly due to different combinations of amino acids or amino alcohols attached at two positions of the central ring (for examples see Fig. 1 A). Thus the function of some (even as yet unknown) enzymes involved in MAA synthesis resembles that of NRPS - they incorporate an amino acid into a larger molecule.

## Conclusive remarks

MAAs and marine toxins show a broad overlap in the taxonomic distribution of their occurrence. They are mainly produced by cyanobacteria and algae, and MAAs as well as toxins are transmitted via food chains to organisms of higher trophic levels. In contrast to the transmission of toxins that causes severe adverse effects on animals, so far there is no evidence for any adverse effects of MAAs on any kind of organism. The biosynthetic pathways of many marine toxins and of MAAs are not fully elucidated but MAAs share the use of amino acids as building blocks with the cyclic peptide toxins of cyanobacteria. Further research on the genes involved in MAA synthesis will help to resolve the question of a possible relationship between NRPS and MAA synthesis.

## Figures and Tables

**Table 1:** General features and taxonomic distribution of the cyanotoxins (modified from [29])

Toxin group^1^	Primary target organ in mammals	Cyanobacterial genera^2^

Cyclic peptides		
Microcystins	Liver	*Microcystis*, *Anabaena*, *Oscillatoria (Planktothrix)*, *Nostoc*, *Hapalosiphon*, *Anabaenopsis*
Nodularin	Liver	*Nodularia*

Alkaloids		
Anatoxin-a	Nerve synapse	*Anabaena*, *Oscillatoria (Planktothrix)*, *Aphanizomenon*
Anatoxin-a(S)	Nerve synapse	*Anabaena*
Saxitoxins	Nerve axons	*Anabaena*, *Aphanizomenon*, *Lyngbya*, *Cylindrospermopsis*
Cylindrospermopsins	Liver^3^	*Cylindrospermopsis*, *Aphanizomenon*, *Umezakia*
Lyngbyatoxin-a	Skin, gastro-intestinal tract	*Lyngbya*
Aplysiatoxins	Skin	*Lyngbya*, *Schizothrix*, *Oscillatoria (Planktothrix)*

Lipopolysaccharides (LPS)	Potential irritant; affects any exposed tissue	All

1 Many structural variants may be known for each toxin group

2 Not produced by all species of the particular genus

3 Whole cells of toxic species elicit widespread tissue damage, including damage to kidney and lymphoid tissue
